# Towards the automated localisation of targets in rapid image-sifting by collaborative brain-computer interfaces

**DOI:** 10.1371/journal.pone.0178498

**Published:** 2017-05-31

**Authors:** Ana Matran-Fernandez, Riccardo Poli

**Affiliations:** School of Computer Science and Electronic Engineering, University of Essex, Colchester, Essex, United Kingdom; National University of Defense Technology College of Mechatronic Engineering and Automation, CHINA

## Abstract

The N2pc is a lateralised Event-Related Potential (ERP) that signals a shift of attention towards the location of a potential object of interest. We propose a single-trial target-*localisation* collaborative Brain-Computer Interface (cBCI) that exploits this ERP to automatically approximate the horizontal position of targets in aerial images. Images were presented by means of the rapid serial visual presentation technique at rates of 5, 6 and 10 Hz. We created three different cBCIs and tested a participant selection method in which groups are formed according to the similarity of participants’ performance. The N2pc that is elicited in our experiments contains information about the position of the target along the horizontal axis. Moreover, combining information from multiple participants provides absolute median improvements in the area under the receiver operating characteristic curve of up to 21% (for groups of size 3) with respect to single-user BCIs. These improvements are bigger when groups are formed by participants with similar individual performance, and much of this effect can be explained using simple theoretical models. Our results suggest that BCIs for automated triaging can be improved by integrating two classification systems: one devoted to target detection and another to detect the attentional shifts associated with lateral targets.

## Introduction

A Brain-Computer Interface (BCI) is a system that allows users to convey commands to interact with external devices using only their thoughts, usually by means of electroencephalography (EEG) signals recorded from their scalp. BCIs were originally conceived with the aim of helping people with severe disabilities, such as those in a complete locked-in state, to communicate [1–4]. However, some BCI systems developed over the last decade have shifted their attention towards able-bodied users, in an attempt to augment human abilities. One type of such systems consists of creating collaborative BCIs (cBCIs) by grouping users (e.g., by fusing information extracted from their individual EEG recordings) with the aim of better controlling an external device or improving their individual performance at a joint task [5–12].

One of the new systems for human augmentation focuses on cortically-coupled vision: the use of a BCI for triaging imagery in order to speed up the detection of images of interest amongst a series of distractors [7, 13–15]. If the ratio of targets vs non-targets is sufficiently low (i.e., around 10%), a P300 Event-Related Potential (ERP) is elicited in response to targets [16], and its detection allows for the classification of images into one of these two categories. Research in this area of application has shown that the rapid presentation of images in the same spatial location (a protocol called Rapid Serial Visual Presentation—RSVP) [17] combined with BCIs can speed up the process of reviewing the images of interest (i.e., reduce triage time) with respect to traditional manual search without detrimental effects on target detection accuracy [18–28]. In the future, these systems could be very useful, for instance, in areas in which large amounts of time-sensitive images need to be reviewed looking for possible targets, as is the case of intelligence analysts.

Accurate and rapid target detection, however, is often only a prerequisite to more sophisticated processing. For instance, techniques such as the one we present in this manuscript, that allows to automatically *locate* targets within the images (a task that cannot be achieved using the P300 ERP alone), could be very beneficial for triage systems.

In previous work [29, 30], we showed that the N2pc is elicited in the conditions of the RSVP paradigm with real aerial images, and that it can be used to discriminate targets depending on ths side of an image where they are located in single-user BCIs. The N2pc is a negative component that generally appears within 170–300 ms of stimulus onset. It can be detected on electrode sites located on the opposite side to the visual field where the target is found, with maximum amplitudes in electrode sites P7/8 and PO7/8 from the 10-20 international system. The maximum amplitudes of the N2pc are around 2–3 *μV* [31–33]. This ERP, which has been widely studied in literature related to attention, is known to be elicited when participants look for a given template in a search display which contains at least one non-target item in addition to the target [31].

The most similar work to the one we report here is that of Putze and collaborators [34], who used EEG data to detect targets and eye tracking to locate them (participants were asked to fixate their eyes on the targets). However, this was not done on an RSVP task with real-world stimuli, but rather on a series of simple stimuli (a number of circles arranged within a larger circle) that were sequentially and randomly flashed for 2 seconds each. While this technique could, in principle, be extended to the real-world stimuli used in this paper, it is unlikely that it could work when images are presented at the high speeds used in RSVP, as there are previous reports of saccades being suppressed at such rates [35, 36].

EEG signals are highly contaminated by noise and artefacts. The usual approach for increasing the signal-to-noise ratio in BCIs, and thus improve their performance, is to average the ERPs recorded over a number of repetitions of each stimulus [37–40]. For example, in their N2pc-driven BCI (which is, to the best of our knowledge, the only system that has used this ERP to control a BCI), Awni and collaborators [41] performed 3 repetitions of each stimulus and averaged across them prior to classifying each trial. One of the drawbacks of this approach is that the increase of performance is obtained by sacrificing speed, which makes this technique impractical for some applications, specially in those designed for able-bodied users. Moreover, averaging across multiple trials is not always possible (e.g., exposing an observer to the same stimulus repeatedly can alter their neural response to it [42, 43]). Combining signals from a number of users via cBCIs in this type of situations, however, has proven to be very useful (e.g., [5, 44]).

The information from multiple participants can be fused at different levels in order to create a collaborative BCI. The simplest method consists of performing averages of the raw EEG recordings from single trials across users and using such averaged data to train a unique classifier for the whole group (i.e., *signal fusion* level). This approach, like the normal averaging across trials that is typical of single-user BCIs, increases the signal-to-noise ratio when the neural responses from the individuals have similar latencies [45–49]. The second level of fusion is the *feature level*, where features are extracted from each user’s EEG. These features can be simply concatenated to form a unique feature vector, or combined in any other way [5, 44], so only one classifier is used (as in the signal level approach). Finally, at the *decision fusion* level, the EEG data from each participant is used to tailor one classifier specifically for him/her, so a decision merging step needs to be implemented [48, 49]. Working at this third level and experimenting with different merging methods, Cecotti and collaborators [48] found that averaging the classifiers’ outputs provided the best performance.

A considerable amount of work has been conducted to establish which level of fusion is optimal, obtaining consistent results across laboratories and applications. In particular, the two approaches that are often compared are single-trial averages across participants (i.e., signal level) and fusion at the decision level (usually averaging classifiers’ outputs to send a command). Since most of the work in that area has been done based on different BCI paradigms, given the inter-subject differences in latencies and amplitudes, it is not surprising that the best performance is obtained when information is merged at the decision level after individually tailoring a classifier for each individual user [5, 48, 49].

Even though it is, in theory, possible to repeat trials for every participant also in the collaborative paradigm, it is expected that the classification will be done in single trials. Indeed, a major advantage of cBCIs is the error correction capability obtained by combining signals, features or decisions across multiple users. In the case of the P300, which is an ERP of relatively big amplitude, across-subject averaging (i.e., merging information at the signal level) is sufficient to provide reliable classification [6, 7], although it is a sub-optimal strategy (with respect to approaches that combine the information at the decision level). However, for smaller ERPs, such as the N2pc, for which variations in latency are small and mostly due to the paradigm used rather than the user [33]), the jury is out as to whether this is or not possible.

With regards to group size, it is typically accepted that increasing group size leads to higher performance [50]. However, it has been shown [51] that this “crowd wisdom” effect is not always present, and that it depends on correlations between the behaviour of the members. In those cases, small groups can maximise accuracy. A study from Bahrami and collaborators [52] provided some evidence that similarity in participants’ behaviour might be more important than group size. According to their results, when observers were paired and given the chance to communicate freely, they performed better if they had similar visual sensitivities [52].

The work presented in this paper extends our previous research along multiple axes. In [29] we reported on the use of the N2pc to approximately locate targets in images that are known to contain one, using single-user BCIs at a presentation rate of 5 Hz. Moreover, by grouping observers into pairs, in [30] we obtained significantly higher accuracies at left vs right classification of targets than with single observers.

The study presented in this paper uses the stimulation protocol and a subset of the participants from [6, 7], where we used 2- and 3-user cBCIs to target *detection*. However, as in [29, 30], in this paper we have applied BCIs to the problem of *localisation of targets* within images (via N2pc ERPs), not to the problem of *classifying* images as containing or not containing a target.

Moreover, here we explore the effects of *selecting* participants in order to form groups in collaborative BCIs, depending on how similar the performance of the group members is. This participant selection method was first presented on [30]. In that work, we showed that pairing participants based on their performance similarity to form cBCIs provided an advantage with respect to forming random pairs. However, we did not study the reasons behind the further improvements that were observed, which we have done here, in addition to extending and validating the model to groups of up to 10 participants.

The paper is organised as follows. In Materials and methods we describe our experimental setup, the signal acquisition and manipulation, the methods used for feature selection and classification, and our participant selection technique. The Results and Discussion sections report and discuss the results of our experiments, respectively, both in terms of ERPs and of the localisation accuracy of our BCI. In these sections we also study the effect of our participant selection method. Finally, we provide some conclusions and indications for future work in the Conclusions section.

## Materials and methods

### Participants and setup

Eleven volunteers with normal or corrected-to-normal vision (with ages ranging between 19–33 years, mean age ± standard deviation = 24.3 ± 3.7 years old, 4 females, 5 left-handed) were initially recruited to participate in our experiment.

The study received the approval from the Ethics Committee of the University of Essex, and consent was obtained from all participants in written form prior to the beginning of the experiment. Recruitment of volunteers was performed via advertising through the University of Essex’s mailing lists in February–March 2013. Only participants above 18 years old were considered for the experiment. Moreover, given the high presentation rates that are used in the RSVP protocol, participants were also screened for any personal or family history of epilepsy. No other exclusion criteria were used. Using these criteria, no participants were excluded from the experiment. All participants completed the experiment and were included in the analysis. No power analysis was performed to calculate sample size.

**Fig 1:**
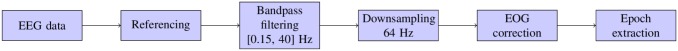
Processing and classification pipeline for the single-user and the collaborative BCIs.

The general pipeline for the data preprocessing and feature extraction steps followed is shown in Fig 1. During the experiment, volunteers were seated at an approximate distance of 80 cm from the screen where the images were presented. EEG signals were collected from 64 ear-referenced channels (following the international 10-20 system) with a BioSemi ActiveTwo system at a sample rate of 2048 Hz. Signals were band-pass filtered from 0.15–28 Hz and downsampled to 64 Hz.

Due to the lack of EOG electrodes, eye blinks and eye movements were removed from the EEG by applying the subtraction algorithm based on correlations [53] to the average of the differences between channels Fp1 and F1 and channels Fp2 and F2.

### Experimental design

Aerial pictures of London, converted to grayscale and with equalised histograms, were shown to participants in sequences (or bursts) of 100 images at presentation rates of 5, 6, and 10 Hz, forming 3 levels of difficulty with 24 bursts each. The 24 bursts of each difficulty level were presented from the lowest to the highest presentation rate. There were no gaps between two consecutive stimuli. Participants could rest between bursts and were free to decide when to start the next burst by clicking on a mouse button.

Picture size was 640 × 640 px^2^ (subtending 11.5° × 11.8° of visual angle).

**Fig 2:**
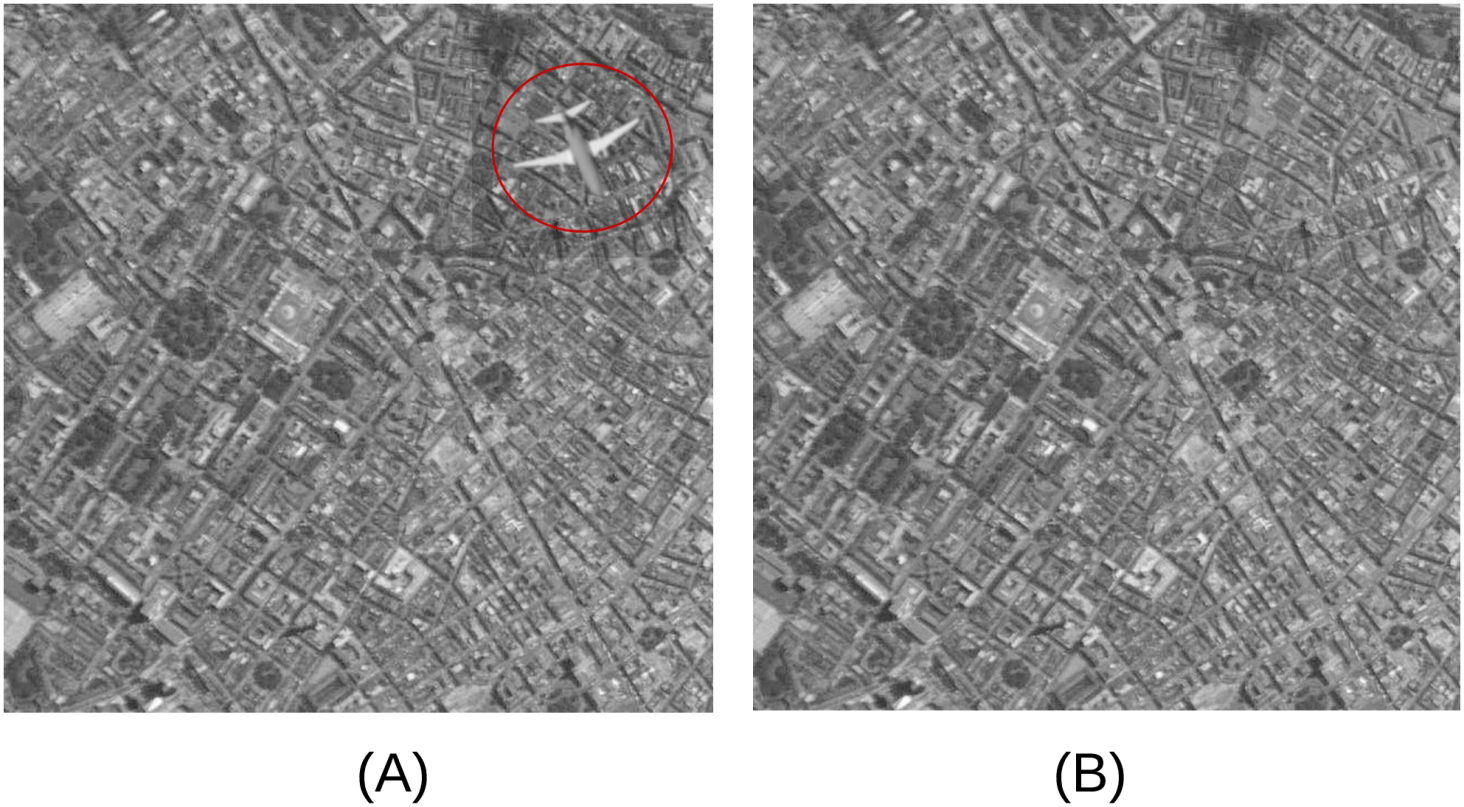
Examples of target and non-target images from our experiment. (A) Target. (B) Distractor. The target has been highlighted for presentation purposes. Satellite imagery for this manuscript was extracted from The Gateway to Astronaut Photography of Earth, image ID ISS040-E-033077. These images are for illustrative purposes only. The images used in the experiment were extracted from Google (Google, BlueSky).

Each burst contained 10 “target” images, each of which contained a randomly rotated and positioned airplane (as exemplified in Fig 2A that was not present in “non-target” images, as illustrated in Fig 2B. In order to guarantee that the targets were completely contained within each target picture, the subfigures were cropped from a large-scale image of London before placing the target in them.

For each target image, we defined the horizontal position of the target as the *x*-coordinate of the centroid of the airplane. Lateral targets were those the centroid of which was positioned at a visual angle of at least ±1.2° on the horizontal axis (with respect to the centre of the screen). Using this criterion, approximately 60% (144 out of 240) of the targets were classed as lateral. Of these, 59 were located on the Left Visual Field (LVF) and 85 on the Right Visual Field (RVF). Targets that were not lateral were classed as central targets.

In order to obtain artefact-free EEG recordings, participants were instructed to try to minimise eye blinks and general movements during the bursts. In order to encourage participants to remain focused on the task, they were asked to mentally count the number of airplanes they saw in each burst, and to verbally report the count of that burst at the end.

### Feature selection and classification

Single-trial EEG epochs containing the time window 200–400 ms referred to picture onset were extracted for each lateral target picture. At a sampling rate of 64 Hz, this results in a total of 14 time samples per channel. These data represent the temporal window where the N2pc is expected to appear, according to the literature [33, 54] and our own previous knowledge [29]. The baseline for epoch referencing was the mean value of the 200 ms interval preceding the onset of the stimulus.

Given the limited number of trials available for the left vs right classification task and the associated potential overfitting risks, we used only four differences between pairs of electrodes (PO7–PO8, P7–P8, PO3–PO4 and O1–O2) [29, 30, 32, 33, 55]. The features extracted for each pair of channel differences were concatenated, yielding a feature-vector representation of 14 × 4 = 56 elements used for classification.

Moreover, we decided to adopt the representation conventions that are typically followed for this ERP component. In particular, the N2pc is usually represented as the difference of the “contralateral” and the “ipsilateral” waveforms, which are defined relative to the position of the target. The contralateral waveform consists of the EEG recordings from electrode sites on the opposite hemisphere to where the object of interest is located, while the ipsilateral waveform consists of recordings from electrodes on the same hemisphere where the target appears. Hence, for an RVF (resp. LVF) target, the contralateral electrodes are those on the left (resp. right) hemisphere, and the ipsilateral electrodes are those on the right (resp. left). In the international 10-20 system, electrode sites on the right hemisphere are represented by even numbers (e.g., PO8, P8), whereas odd numbers correspond to electrodes on the left side (e.g., PO7, P7).

#### Individual classification

In order to assess whether the collaborative BCI approach yields an advantage with respect to single-user BCIs, we first focused on the single-trial, single-user discrimination between LVF and RVF targets (i.e., left vs right classification) of lateral target images.

We measured the performance of the individual BCI systems through a double cross-validation loop. The outer loop divided the epochs in our dataset into a training and a test set, containing 75% and 25% of the data, respectively. This train-test split was randomly performed 10 times with replacement. Although we did not allow for resampling within a split (i.e., an epoch could not appear multiple times in the same training set), since the split was performed 10 times and the training set contained 75% of the dataset, the data were randomly resampled between splits. Each pass of the outer cross-validation loop contained an inner 10-fold stratified cross-validation loop. The training set of the inner loop was used to find the optimal C value for a linear-kernel SVM classifier for each participant.

As we will explain below, the mean performance of the participants in the test set of the inner cross-validation loop was used to determine whether they should be included or not in a group according to our similarity-based selection method. The test set of the outer loop was used as an independent set of data for N2pc detection.

#### Collaborative classification

**Fig 3:**
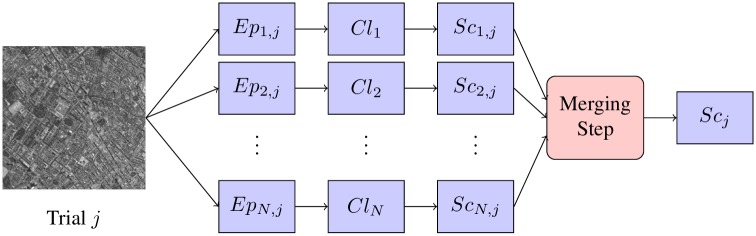
Classification and merging steps for the MC-cBCI and LDA-cBCI ways of creating a collaborative BCI. Epochs corresponding to trial *j* are extracted from all the group members, *Ep*_*i*,*j*_, *i* ∈ [1, *N*], where *N* is the group size. Each participant *i* has an individually tailored classifier, *Cl*_*i*_. The outputs of the *N* classifiers in response to the individual epochs, *Sc*_*i*,*j*_ are merged either by averaging the scores (MC-cBCI approach) or through an LDA classifier (LDA-cBCI approach) in order to obtain a final group score, *Sc*_*j*_, which is used to measure the group performance by means of the AUC.

We used three methods to merge signals from multiple participants in order to create collaborative BCIs: (1) working at the signal fusion level, we averaged the feature vectors for each trial across group members and trained a unique classifier for each group (Single Classifier cBCI, SC-cBCI); (2) at the decision fusion level, where each participant has his/her own individually tailored classifier, we calculated the final output as the mean from the individuals’ outputs for each trial, creating a Multiple Classifier cBCI (MC-cBCI); or, also at the decision level of fusion, (3) we trained a Linear Discriminant Analysis (LDA) classifier to merge the outputs of the individual classifiers and make a decision (LDA-cBCI). Fig 3 shows the pipeline for classifying a trial in the MC-cBCI and LDA-cBCI approaches.

While the MC-cBCI approach (averaging classifier outputs) assigns the same weight to each participant, the LDA-cBCI approach is a sort of weighed voting, so that those participants that perform better may be given a higher weight.

The analogue outputs of the classifiers were recorded and used to compute the Receiver Operating Characteristic (ROC) curve for each participant. The performance of the classifiers was then assessed by condensing the information from the ROC curve into the Area Under the ROC Curve (AUC) [56, 57].

#### Group-member selection

The group-member selection method presented here is based on the creation of groups according to the similarity in performance of the participants (i.e., their individual AUCs). We tabulated different levels (or thresholds) of similarity *δ*, based on the difference between the maximum and the minimum AUCs across the participants. We term this difference the *dissimilarity index*. More specifically, a set of participants *R* is allowed to form a group if the dissimilarity index of the group was below a threshold *δ*. That is, the group could be formed if

$$\begin{array}{c} \max_{x\in R}\left(AUC_{x}^{f}\right)-\min_{x}\left(AUC_{x}^{f}\right)\leq \delta , \end{array}$$

where $AUC_{x}^{f}$ represents the AUC value for participant *x* (with *x* = 1, …, 11) at the presentation rate *f* (with *f* = 5, 6, 10 Hz).

In order to assess the influence of similarity of group members on cBCI performance, the threshold *δ* was set at 5%, 10%, …, 25% and only the cBCIs obtained from groups of subjects for which the dissimilarity index was below the threshold were considered. For comparison, we also included the situation where no group selection was performed (i.e., *δ* = 100%).

#### Collaborative target localisation

For each participant, we also used the 14 samples from each of the four pairs of differences between contralateral electrodes (PO7–PO8), (P7–P8), (PO3–PO4) and (O1–O2) as inputs to train (through cross-validation) a Neural Network (NN) to predict the horizontal position of targets within images. The training set of each fold was used to find the optimal number of neurons of the hidden layer (5, 10 or 20) and their activation function (hyperbolic tangent or sigmoid). The output neuron was linear.

We then created a cBCI which optimally combined the outputs of individual NN regressors. This was achieved using an LDA regressor, hence assigning different weights to the different group members when making the prediction of the location of the target.

The neural networks were trained using only lateral targets. However, in the Results section we will also show how the target localisation method works for targets that were in the centre of the screen.

## Results

We start this section by looking at the shape and characteristics of the N2pc ERP that is elicited in our experiments. Then we will address the matter of the performance of single-user BCIs (sBCIs) and cBCIs for the left vs right single-trial classification of targets in images that are known to contain one, and a theoretical analysis of the reasons behind the improvements that are obtained by the cBCI over the sBCIs. Finally, we will evaluate the degree to which the outputs of the neural network can predict the position of a target in an image, both in the single-user and the collaborative cases.

### ERP analysis

As previously stated (see Feature selection and classification section), we decided to follow the conventions from the literature and represent the N2pc as the difference between the contralateral and ipsilateral waveforms across all lateral-target epochs from the training set of one of the cross-validation folds. Furthermore, also following the representation conventions for this ERP, we used an *inverted ordinate* axis, so higher means more negative.

**Fig 4:**
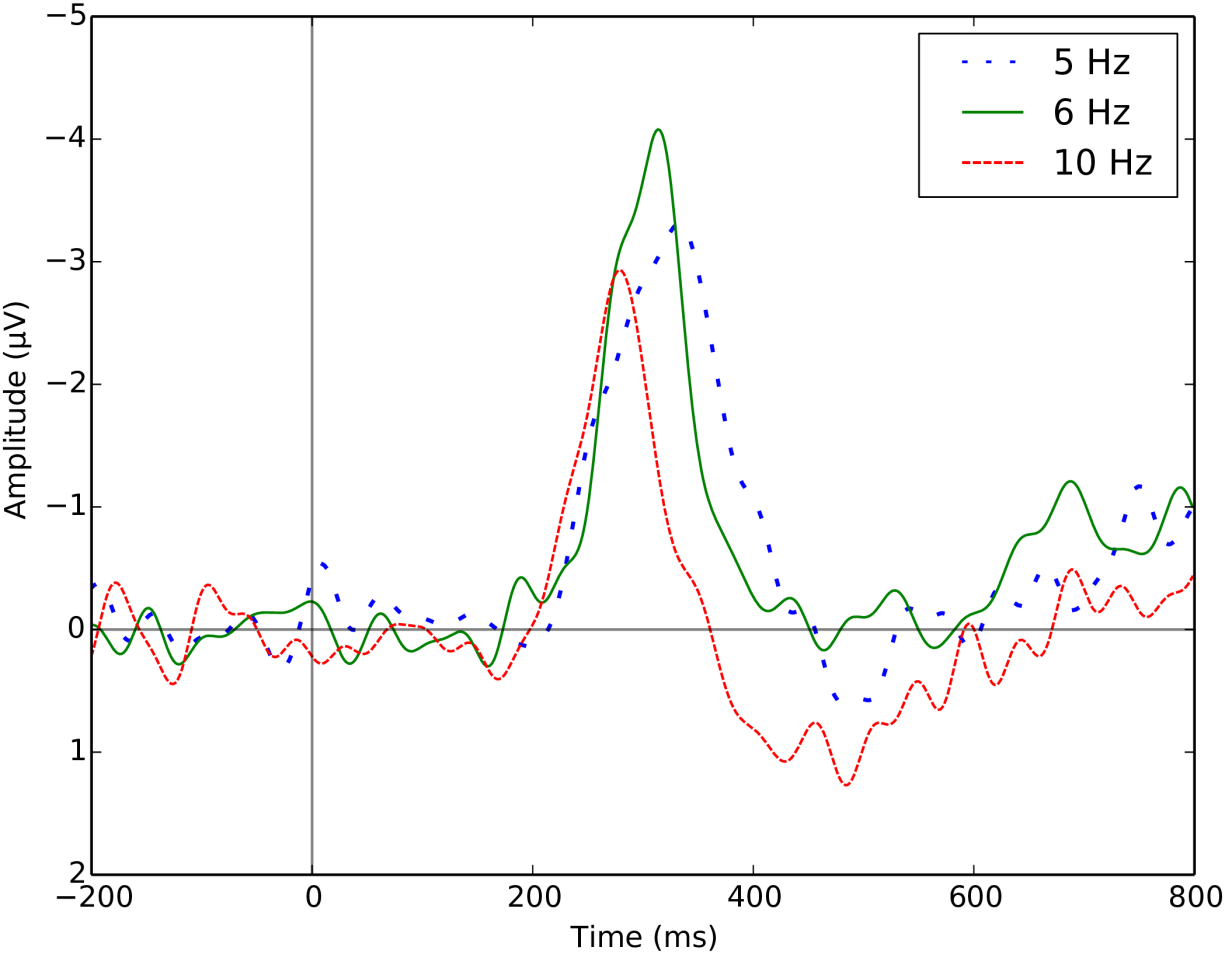
Grand-averaged difference plot of the contralateral minus the ipsilateral waveforms recorded at electrode sites PO7 and PO8 across lateral targets from the training set of one split.

Fig 4 shows the grand-averages for the N2pc, for different presentation rates, measured at electrode sites PO7 and PO8. The shape and timing of the N2pc ERPs shown in our grand-average difference plots are consistent with those reported in the literature [31–33]. However, we see in this figure two interesting effects: (1) the latency of the N2pc (measured as the time when the difference waveform reaches its peak) tends to become shorter as the presentation rate increases, and (2) the peak amplitude at a presentation rate of 10 Hz is the smallest of the three tested.

We measured peak amplitudes and tested for statistical differences across the three conditions using an univariate Mann-Whitney U test. The N2pc that is elicited at 10 Hz, with a peak amplitude of -1.12 *μV*, is significantly smaller than for lower rates (*p* = 1.9 × 10^−11^ for 5 vs 10 Hz, and *p* = 6.7 × 10^−8^ for 5 vs 6 Hz, after Bonferroni correction). There were no statistical differences in peak amplitudes between the presentation rates of 5 (peak amplitude = -2.51 *μV*) and 6 Hz (peak amplitude = -2.45 *μV*).

There are at least three possible reasons for these rate-related changes, and they are not mutually exclusive: (1) the target detection task is harder for participants at high presentation rates due to the shorter duration of the target stimuli; (2) the average temporal distance between consecutive targets decreases as the stimulation rate increases, causing some targets to fall within a possible “refractory period” for the N2pc, such as those associated with repetition blindness and the attentional blink [58–61]; and (3) our choice of experimental desig. Since participants become progressively more and more tired as the experiment progresses, with an associated drop in attention levels, it is possible that they consequently missed more targets than in previous difficulty levels. We elaborate on these factors below and in the Discussion section.

#### Task difficulty

Evidence indicating that the difficulty of the task increases is provided by our records of the plane counts reported by the participants for every burst. Indeed, as shown in Table 1, average plane counts decrease as the presentation rate increases. Since participants did not have time to foveate to targets (especially for the fastest presentation rate), it is possible that those positioned laterally were missed more frequently than those presented in the centre of the screen. Since grand averages do not take into account which lateral targets were seen and which were not, the amplitude of the N2pc component might have been artificially reduced due to the high percentage of missed targets.

**Table 1:** Average total plane counts reported by participants as a function of presentation rate.

	5 *Hz*	6 *Hz*	10 *Hz*
Average plane count	197.2	186.7	157.2
Sensitivity	82.2%	78.8%	65.5%

There were 240 airplanes in total in each level of difficulty.

#### Refractory period of the N2pc

**Fig 5:**
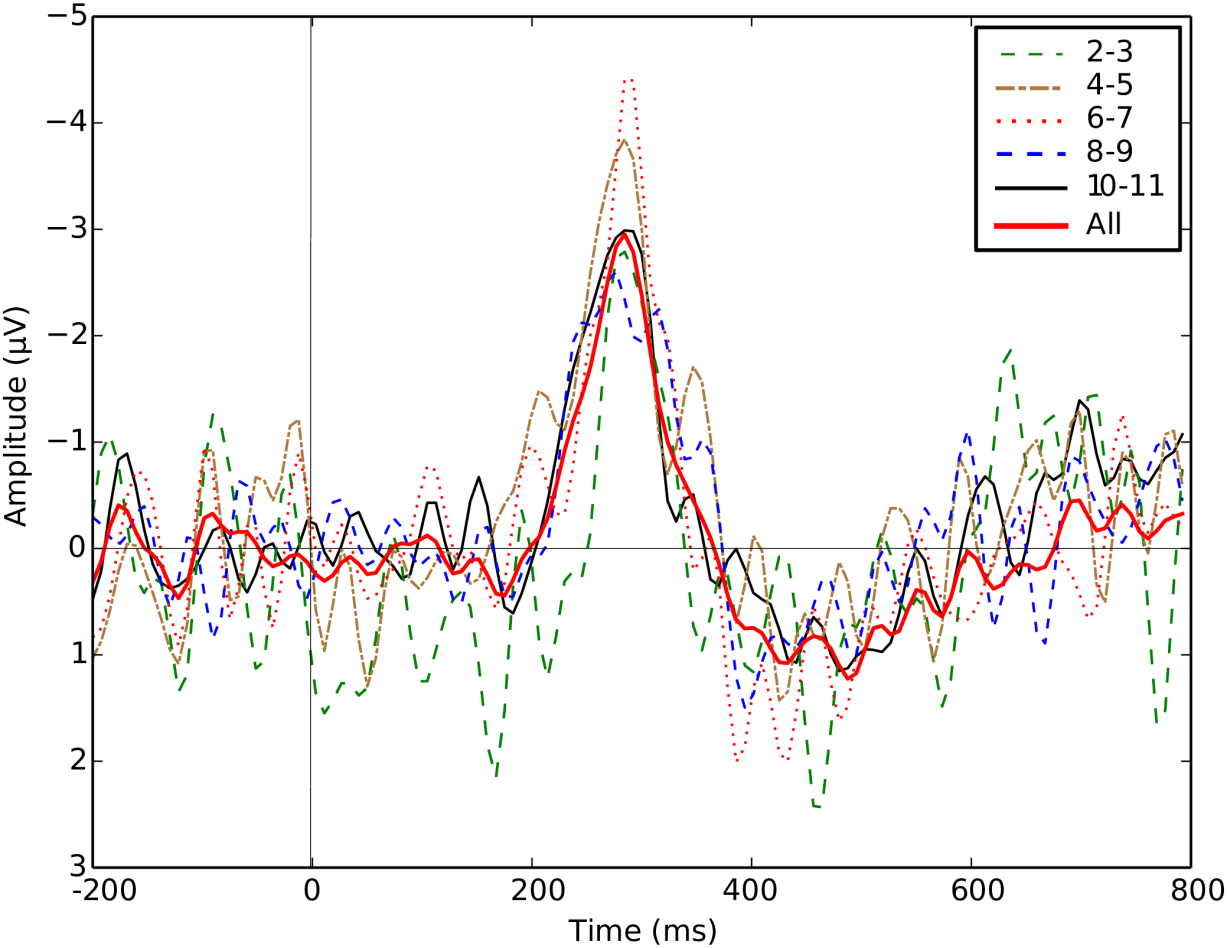
Grand-averaged contralateral minus ipsilateral waveforms recorded at PO7 and PO8 across all epochs from the training set of one split for a presentation rate of 10 Hz, separated depending on the distance (in number of images) to the previous target.

Repetition blindness and the attentional blink have been shown to play a role in other ERP-based BCIs, such as those based on the P300 [62]. These phenomena manifest themselves as a participant missing a target when the separation from a previous target is less than 500 ms. To test whether some form of refractory period was influencing the ERP amplitudes, the epochs were divided and analysed on the basis of the number of non-targets separating two targets. Fig 5 shows grand averages of the N2pc (again, plotted as the contralateral minus the ipsilateral waveforms and using an inverted ordinate axis), for targets that are 2–3, 4–5, 6–7, 8–9 and 10–11 stimuli away from the previous target, for the presentation rate of 10 Hz. There are 70, 140, 150, 220 and 350 epochs of each kind (across all participants), respectively.

At a presentation rate of 10 Hz, the N2pc ERPs associated with well-separated targets (e.g., the line labelled as 10–11) are *not* significantly bigger than the N2pc’s for poorly separated targets, i.e., line 2–3 in the figure. Indeed, *p* = 0.27 for a one-sided Mann-Whitney U test comparing peak amplitudes of lateral targets that are separated by less than 300 ms—i.e. those labelled as “2–3” in Fig 5—vs the rest, for all samples in the interval 264–307 ms. This suggests that refractory phenomena like repetition blindness and the attentional blink may not be responsible for the presentation-rate modulations of the N2pc observed.

#### Experimental paradigm

Another possible explanation for the differences in N2pc amplitudes and latencies is that they could partly be attributed to tiredness and learning effects. This is a possibility as the order of the conditions across subjects was not randomised. We excluded randomisation after receiving early feedback that suggested that participants with no previous experience of high-speed RSVP protocols (such as our cohort) found it exceptionally taxing to start with the 10 Hz condition. Due to this design decision, we cannot exclude the possibility that some of the observed differences in N2pc amplitudes and latencies for different presentation rates are associated with presentation order effects.

Now that we have established that the N2pc is present in the conditions of our experiments, we will focus on the performance of our classifiers for left vs right discrimination of lateral targets in single-user and collaborative BCIs.

### Single-user left vs right classification

Table 2 reports the mean AUC values obtained individually for each participant in left vs right classification using single-trial sBCIs for each presentation rate. These values were obtained, for each participant, across all test sets of the inner cross-validation loop. The table also includes the median AUCs across all participants and test sets of the outer loop. The last row reports the *p* value of a two-sided paired Mann-Whitney U test comparing each individual’s average performance in the inner cross-validation loop with his/her performance in the outer loop, showing that there are no significant differences between them even *before* Bonferroni correction (i.e., *p* > 0.05 for all presentation rates).

**Table 2:** Cross-validation mean AUC values and corresponding standard deviations obtained by our sBCI for LVF vs RVF classification for each participant at different presentation rates.

Participant	5 Hz	6 Hz	10 Hz
1	0.78 ± 0.04	0.85 ± 0.03	0.80 ± 0.04
2	0.85 ± 0.03	0.91 ± 0.02	0.80 ± 0.03
3	0.78 ± 0.05	0.85 ± 0.02	0.83 ± 0.03
4	0.80 ± 0.04	–	0.60 ± 0.08
5	0.82 ± 0.04	0.82 ± 0.03	0.82 ± 0.04
6	0.66 ± 0.04	0.56 ± 0.03	0.55 ± 0.03
7	0.64 ± 0.06	0.76 ± 0.04	0.71 ± 0.03
8	0.61 ± 0.06	0.68 ± 0.04	0.60 ± 0.05
9	0.77 ± 0.04	0.73 ± 0.03	0.83 ± 0.04
10	0.66 ± 0.04	0.66 ± 0.05	0.55 ± 0.04
11	0.71 ± 0.06	0.76 ± 0.03	–
**Median**	**0.77**	**0.76**	**0.75**
**Median on** ***test*** **set**	**0.77**	**0.77**	**0.72**
***p* value**	0.79	0.12	0.28

Empty spaces are due to problems in the data recording for some participants and presentation rates.

While there are clearly large performance variations across participants in the table (similarly to [29, 41]), the AUC medians are reasonably high [63]. Overall, classification results indicate that target localisation by means of the N2pc is possible in the conditions of our experiments.

Taking into account the ERP plots in Fig 4 and the significant differences in amplitude between the 6 Hz and the 10 Hz conditions, we expected performance to drop markedly at 10 Hz. However, the differences in performance observed in Table 2 for the different presentation rates are not statistically significant.

It is worth noting that performance for most participants is well above that of a random classifier (i.e., AUC = 0.5) and that the top quartile of our participants have AUCs ≥ 0.80. This suggests that with a suitable participant selection process a BCI for LVF vs RVF classification BCI could also be successfully operated at high rates.

### Collaborative left vs right classification

Before we look at the results obtained by our collaborative BCIs, we begin this section by quantifying the effect that our participant selection method has on the number of possible groups that can be formed. When no participant selection is applied, with *N* participants, we can form (Nr) distinct groups of size *r*. However, when selecting groups based on performance similarity, the number of groups is smaller. Table 3 reports the effects that different values of the dissimilarity-index threshold *δ* (see Group-member selection section) have on the fraction of groups that can be accepted for the presentation rate of 6 Hz. Obviously, all groups are accepted for *δ* = 100%, so this case is not reported in the table.

**Table 3:** Percentages of groups that are accepted by our selection mechanism for different group sizes and the dissimilarity-index threshold *δ*, for the presentation rate of 6 Hz.

	Group size							
*δ*	2	3	4	5	6	7	8	9
5%	15%	1%	0%	0%	0%	0%	0%	0%
10%	48%	16%	3%	0%	0%	0%	0%	0%
15%	64%	32%	12%	3%	0%	0%	0%	0%
20%	82%	61%	43%	28%	16%	7%	2%	0%
25%	88%	72%	54%	38%	23%	12%	4%	0%

The low percentages of accepted groups that are seen for large group sizes are due to bigger spread of AUCs, so fewer groups can be accepted for a particular threshold.

Table 4 shows the median AUC values that are obtained for a presentation rate of 6 Hz, separately for the three types of collaborative BCIs and for different values of the threshold *δ*, on the test set of the outer loop. Comparing this table with the median AUCs obtained in single-user BCIs reported on Table 2, it can be seen that AUCs are markedly higher for collaborative BCIs than for single-user BCIs. Generally, performance of cBCIs decreases with increasing dissimilarity indices, but, even when no participant selection is performed (i.e., *δ* = 100%), on average, cBCIs are better than the corresponding single-user BCI. For any particular level of performance one may want to achieve, say an AUC of 0.95, one can see in the table the benefit of our group selection strategy, that is, the smaller *δ* the smaller the group required to achieve the target level of performance. So, by selecting groups, we can use much smaller groups to achieve a particular AUC.

**Table 4:** Median AUC values for our types of cBCIs for left vs right classification, as a function of the dissimilarity-index threshold *δ*, for a presentation rate of 6 Hz.

		Group size								
Method	*δ*	2	3	4	5	6	7	8	9	10
SC-cBCI	5%	0.84	0.90	–	–	–	–	–	–	–
	10%	0.87	0.91	0.94	0.97	–	–	–	–	–
	15%	0.87	0.90	0.93	0.95	0.96	–	–	–	–
	20%	0.86	0.89	0.91	0.93	0.95	0.95	0.95	–	–
	25%	0.85	0.88	0.91	0.94	0.95	0.96	0.97	–	–
	100%	0.84	0.87	0.90	0.92	0.93	0.95	0.96	0.96	0.97
MC-cBCI	5%	0.84	0.92	–	–	–	–	–	–	–
	10%	0.87	0.92	0.95	0.97	–	–	–	–	–
	15%	0.86	0.91	0.95	0.96	0.98	–	–	–	–
	20%	0.86	0.91	0.93	0.95	0.96	0.96	0.97	–	–
	25%	0.86	0.91	0.93	0.95	0.96	0.97	0.98	–	–
	100%	0.85	0.89	0.92	0.94	0.95	0.96	0.97	0.97	0.98
LDA-cBCI	5%	0.84	0.92	–	–	–	–	–	–	–
	10%	0.87	0.93	0.95	0.97	–	–	–	–	–
	15%	0.86	0.92	0.95	0.97	0.98	–	–	–	–
	20%	0.86	0.91	0.94	0.95	0.96	0.96	0.97	–	–
	25%	0.86	0.91	0.93	0.95	0.96	0.97	0.98	–	–
	100%	0.86	0.90	0.92	0.94	0.95	0.96	0.96	0.97	0.97

For a deeper analysis of the degree to which a cBCI provides improvements over individual sBCI performance, we compared the results of applying group selection in this target localisation system to the results obtained with two other reference systems for making joint decisions: (1) one unintelligent system that chooses a random member of a group to follow the classification decisions provided by the sBCI associated with him/her, and (2) one, more intelligent, system that always chooses the decisions of the better performing individual in a group. Obviously, the AUCs obtained in these two systems would be, for a group of size *r*, the avg(*AUC*_1_, *AUC*_2_, …, *AUC*_*r*_) for the former, and max(*AUC*_1_, *AUC*_2_, …, *AUC*_*r*_) for the latter, where *AUC*_*i*_ represents the AUC of the sBCI adapted to group member *i* = 1, 2, …, 11.

It should be noted that the selection of participants to form a group is based on their individual performance on the *inner* loop. However, in order to compare the performance of each of these two decision strategies with the performance of the cBCI we used the AUC obtained by each group on the test sets of the *outer* loop.

#### Comparison of the cBCI with the average group member

Table 5 reports the median changes in AUC over the average performance of the individuals in each group for the presentation rate of 6 Hz, separately for the three types of collaborative BCIs—single-classifier cBCIs, multiple-classifier cBCIs and LDA-based cBCIs—for different values of the dissimilarity-index threshold *δ*. Values in bold face are statistically significantly superior (or inferior if preceded by a negative sign) at the 5% confidence level according to a Bonferroni-corrected two-sample one-sided Kolmogorov-Smirnov test comparing the performance of the group vs the average AUC of the participants that form that group.

**Table 5:** Median changes in performance with respect to the average participant of the group at a presentation rate of 6 Hz, as a function of group size and the dissimilarity-index threshold *δ*.

		Group size								
Method	*δ*	2	3	4	5	6	7	8	9	10
SC-cBCI	5%	**+4.0%**	+8.4%	–	–	–	–	–	–	–
	10%	**+3.9%**	**+9.2%**	**+12.9%**	+15.9%	–	–	–	–	–
	15%	**+4.8%**	**+10.1%**	**+12.5%**	**+15.7%**	+16.3%	–	–	–	–
	20%	**+4.8%**	**+10.4%**	**+13.7%**	**+16.1%**	**+18.1%**	**+19.5%**	+20.1%	–	–
	25%	**+5.6%**	**+10.3%**	**+13.6%**	**+16.1%**	**+17.9%**	**+19.3%**	+20.0%	–	–
	100%	**+6.1%**	**+10.9%**	**+14.5%**	**+17.0%**	**+18.6%**	**+20.4%**	**+21.7%**	**+22.6%**	+23.5%
MC-cBCI	5%	**+7.4%**	+12.8%	–	–	–	–	–	–	–
	10%	**+7.0%**	**+12.2%**	**+15.5%**	+18.0%	–	–	–	–	–
	15%	**+7.3%**	**+12.2%**	**+15.2%**	**+17.5%**	+19.1%	–	–	–	–
	20%	**+7.2%**	**+12.3%**	**+15.7%**	**+18.4%**	**+19.9%**	**+22.1%**	+23.0%	–	–
	25%	**+7.2%**	**+12.3%**	**+15.9%**	**+18.3%**	**+19.7%**	**+21.3%**	+21.9%	–	–
	100%	**+7.3%**	**+13.2%**	**+17.0%**	**+19.9%**	**+21.8%**	**+23.5%**	**+25.1%**	**+25.8%**	+26.4%
LDA-cBCI	5%	**+7.4%**	+12.6%	–	–	–	–	–	–	–
	10%	**+7.4%**	**+12.6%**	**+15.5%**	+18.6%	–	–	–	–	–
	15%	**+7.4%**	**+12.8%**	**+15.9%**	**+18.1%**	+19.8%	–	–	–	–
	20%	**+7.5%**	**+12.8%**	**+16.3%**	**+18.9%**	**+20.7%**	**+22.5%**	+23.3%	–	–
	25%	**+7.7%**	**+12.9%**	**+16.3%**	**+18.5%**	**+20.3%**	**+21.2%**	+21.8%	–	–
	100%	**+8.0%**	**+14.2%**	**+18.1%**	**+20.5%**	**+22.4%**	**+23.9%**	**+24.9%**	**+25.4%**	+26.0%

Values in bold face are statistically significantly superior at the 5% confidence level according to a two-sample one-sided Kolmogorov-Smirnov test comparing the performance of the groups vs the average AUC within those groups (after Bonferroni correction).

All values in the table are positive, indicating that, irrespective of the dissimilarity-index threshold *δ* and stimulation frequency, cBCIs produce better AUCs than the unintelligent system that randomly picks the responses of an individual in a group. These improvements are statistically significant at a presentation rate of 6 Hz for all values of *δ* and all types of cBCIs.

Similar results were obtained for the presentation rates of 5 and 10 Hz, with gains and pattern of behaviour very close to those reported in Table 5.

#### Comparison of the cBCI with the best member of the group

We now place ourselves in the much more challenging scenario represented by the second reference system: comparing the performance of a cBCI with that of the best participant of each group. Table 6 reports the median changes in performance over the best participant of each group for the presentation rate of 6 Hz, for different values of the dissimilarity-index threshold *δ*.

**Table 6:** Median changes in performance with respect to the best participant of the group at a presentation rate of 6 Hz, as a function of group size and the dissimilarity-index threshold *δ*.

		Group size								
Method	*δ*	2	3	4	5	6	7	8	9	10
SC-cBCI	5%	*+2.9%*	+5.7%	–	–	–	–	–	–	–
	10%	**+1.8%**	**+4.7%**	**+6.3%**	+6.8%	–	–	–	–	–
	15%	**+1.6%**	**+3.8%**	**+5.8%**	**+7.0%**	+5.9%	–	–	–	–
	20%	**+0.3%**	**+2.3%**	**+3.4%**	**+4.9%**	**+5.5%**	**+5.7%**	+4.9%	–	–
	25%	**+0.0%**	**+1.3%**	**+3.3%**	**+4.3%**	**+5.7%**	**+6.0%**	+6.6%	–	–
	100%	-0.8%	**+0.0%**	**+1.3%**	**+2.2%**	**+3.4%**	**+4.3%**	**+5.2%**	**+5.6%**	+5.8%
MC-cBCI	5%	**+4.2%**	+10.1%	–	–	–	–	–	–	–
	10%	**+3.6%**	**+7.5%**	**+9.0%**	+8.7%	–	–	–	–	–
	15%	**+2.9%**	**+5.6%**	**+8.4%**	**+8.7%**	+8.5%	–	–	–	–
	20%	**+1.2%**	**+3.8%**	**+5.1%**	**+6.1%**	**+7.3%**	**+7.7%**	+7.5%	–	–
	25%	**+0.5%**	**+3.5%**	**+5.1%**	**+6.2%**	**+7.3%**	**+8.0%**	+8.3%	–	–
	100%	**-0.3%**	**+2.0%**	**+3.5%**	**+4.7%**	**+5.7%**	**+6.9%**	**+7.4%**	**+8.0%**	+8.4%
LDA-cBCI	5%	**+4.7%**	+10.0%	–	–	–	–	–	–	–
	10%	**+3.8%**	**+7.0%**	**+9.2%**	+9.3%	–	–	–	–	–
	15%	**+3.0%**	**+6.4%**	**+8.9%**	**+8.9%**	+9.2%	–	–	–	–
	20%	**+1.4%**	**+4.0%**	**+5.1%**	**+7.0%**	**+7.8%**	**+8.1%**	+7.8%	–	–
	25%	**+1.0%**	**+3.6%**	**+5.1%**	**+6.7%**	**+7.8%**	**+8.1%**	+8.1%	–	–
	100%	**+0.6%**	**+3.2%**	**+4.2%**	**+5.0%**	**+6.1%**	**+6.8%**	**+7.3%**	**+7.9%**	+8.0%

Values in bold face are statistically significantly superior at the 5% confidence level according to a two-sample one-sided Kolmogorov-Smirnov test comparing the performance of the groups vs the maximum AUC within those groups (after Bonferroni correction).

In virtually all cases, all types of collaborative BCIs outperform the best performer of the corresponding group. Again, most of the values in the table are significantly superior according to a two-sample one-sided Kolmogorov-Smirnov test comparing the performance of the groups vs the AUC of the best performer of the group (after Bonferroni correction), indicating that cBCIs tend to produce better AUCs than the “intelligent” reference system too. In this case, however, we see a dependency of performance on *δ*, with the smaller the *δ*, the bigger the gain of a cBCI over the reference system.

As in the case of comparing the results to the average group performer, similar results to those from Table 6 were obtained also for the 5 and 10 Hz presentation rates.

**Fig 6:**
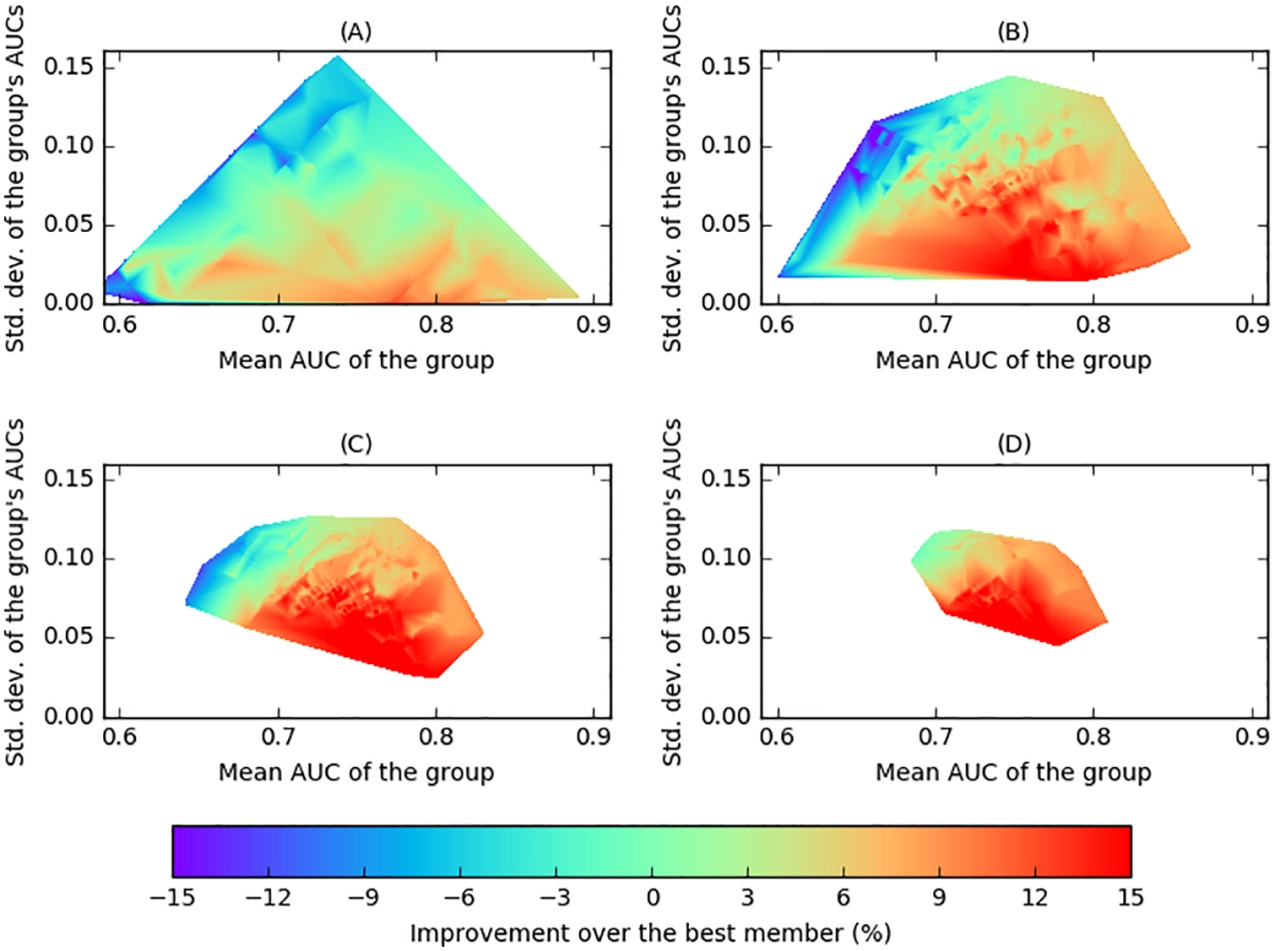
Surface interpolation of the changes in the AUC (in percentage) over the AUC of the best member of the group, for different group sizes. (A) Size 2. (B) Size 4. (C) Size 6. (D) Size 8. The changes are calculated over the AUC of the best participant of a group, and plotted with respect to the mean (horizontal axis) and standard deviation (vertical axis) of the individual AUCs of that group.

The dependency of performance on *δ* is further illustrated in Fig 6, which shows graphically a linear interpolation of the changes in AUC obtained by the MC-cBCI over the best individual AUC in each of the groups of sizes 2, 4, 6, and 8, across all stimulation frequencies. The horizontal and vertical axes of each figure represent the mean and standard deviation of the AUCs of the groups, respectively. Thus, groups of similar participants (i.e., low standard deviation in the group’s AUCs) are located in the lower part of each plot. The figures show quite clearly that improvements in performance tend to be associated with higher similarity between the participants’ AUCs, and that they are relatively independent of the mean AUC of the group.

### Theoretical analysis

In this section we will use a simple theoretical analysis to more formally explain the reasons for the higher improvements in performance that are obtained by the cBCI over the corresponding single-user BCIs when groups are formed taking into account the similarity of the performance of the individuals.

The AUC for each participant can be interpreted as a measure of how spread and separated the distributions of scores for each class are. The bigger the overlap in these distributions, the lower the AUC value and *vice versa*.

As we have previously explained, the MC-cBCI method consists of averaging classifiers’ outputs to obtain the AUC of the cBCI for each group of participants. If we first focus on groups of size 2, the distribution of the average of two *uncorrelated* stochastic variables is the convolution of their pdfs (save for a scaling factor). Formally, let *S*_*i*,*c*_ be a stochastic variable representing the scores produced by a classifier for class *c* ∈ {*C*_1_, *C*_2_} and participant *i* = 1, 2, …, 11, and let *pdf*_*i*,*c*_(*x*) be its probability density function. Here, *C*_1_ and *C*_2_ are classes *L* and *R*, respectively, for LVF vs RVF classification. Then, the pdf of the average of the scores for participants *i* and *j* when presented with a stimulus of class *c*, *S*_*i*,*j*,*c*_ = (*S*_*i*,*c*_ + *S*_*j*,*c*_)/2, is given by ${\text{pdf}}_{i,j,c}\left(x\right)=({\text{pdf}}_{i,c}*{\text{pdf}}_{j,c})(\frac{x}{2})$, where * is the convolution operator.

For simplicity, let us assume that the variables *S*_*i*,*c*_ are normally distributed, i.e., $S_{i,c}∼N\left(\mu_{i,c},\sigma_{i,c}^{2}\right)$. Because the convolution of two Gaussians is a Gaussian, we have that also $S_{i,j,c}∼N\left(\mu_{i,j,c},\sigma_{i,j,c}^{2}\right)$ with

$$\begin{array}{c} \mu_{i,j,c}=\frac{\mu_{i,c}+\mu_{j,c}}{2}\;\;\;\;\text{and}\;\;\;\;\sigma_{i,j,c}^{2}=\frac{\sigma_{i,c}^{2}+\sigma_{j,c}^{2}}{4}. \end{array}$$

Let us further assume that all participants have the same means for the two classes, i.e., *μ*_*i*,*C*_1__ = *μ*_*C*_1__ and *μ*_*i*,*C*_2__ = *μ*_*C*_2__, for *i* = 1, …, 11, and that the standard deviations for the classes are identical, i.e., *σ*_*i*,*C*_1__ = *σ*_*i*,*C*_2__ = *σ*_*i*_ (but not the same for each participant). In this case, we have that

$$\begin{array}{c} \mu_{i,j,c}=\mu_{c}\;\;\;\;\text{and}\;\;\;\;\sigma_{i,j,c}^{2}=\sigma_{i,j}^{2}=\frac{\sigma_{i}^{2}+\sigma_{j}^{2}}{4}. \end{array}$$

That is, the mean becomes independent from the pair (*i*, *j*) that forms the group, and the standard deviation is independent from the class, but depends on the (*i*, *j*) pair.

The separation between the distributions of scores jointly produced by a pair of participants can then be compared with the separation between the distributions of scores of the better performer from the pair. To do this, given the aforementioned assumptions, only the group’s variance, $\sigma_{i,j}^{2}$, needs to be compared against the variance of the better participant of the group, which can be obtained as $\sigma_{min}^{2}=\min\left(\sigma_{i}^{2},\sigma_{j}^{2}\right)$. Since the means of the distributions for classes *C*_1_ and *C*_2_ remain constant, the AUC (calculated from the pdfs of the distributions) of the group will be better than that of the better participant when $\sigma_{i,j}^{2}<\sigma_{min}^{2}$. If we estimate the parameters of the distributions (i.e., means and standard deviations) from real data, the theory presented here allows calculating the AUCs from the pdfs of *C*_1_ and *C*_2_, so it is possible to compute the expected gains/losses.

**Fig 7:**
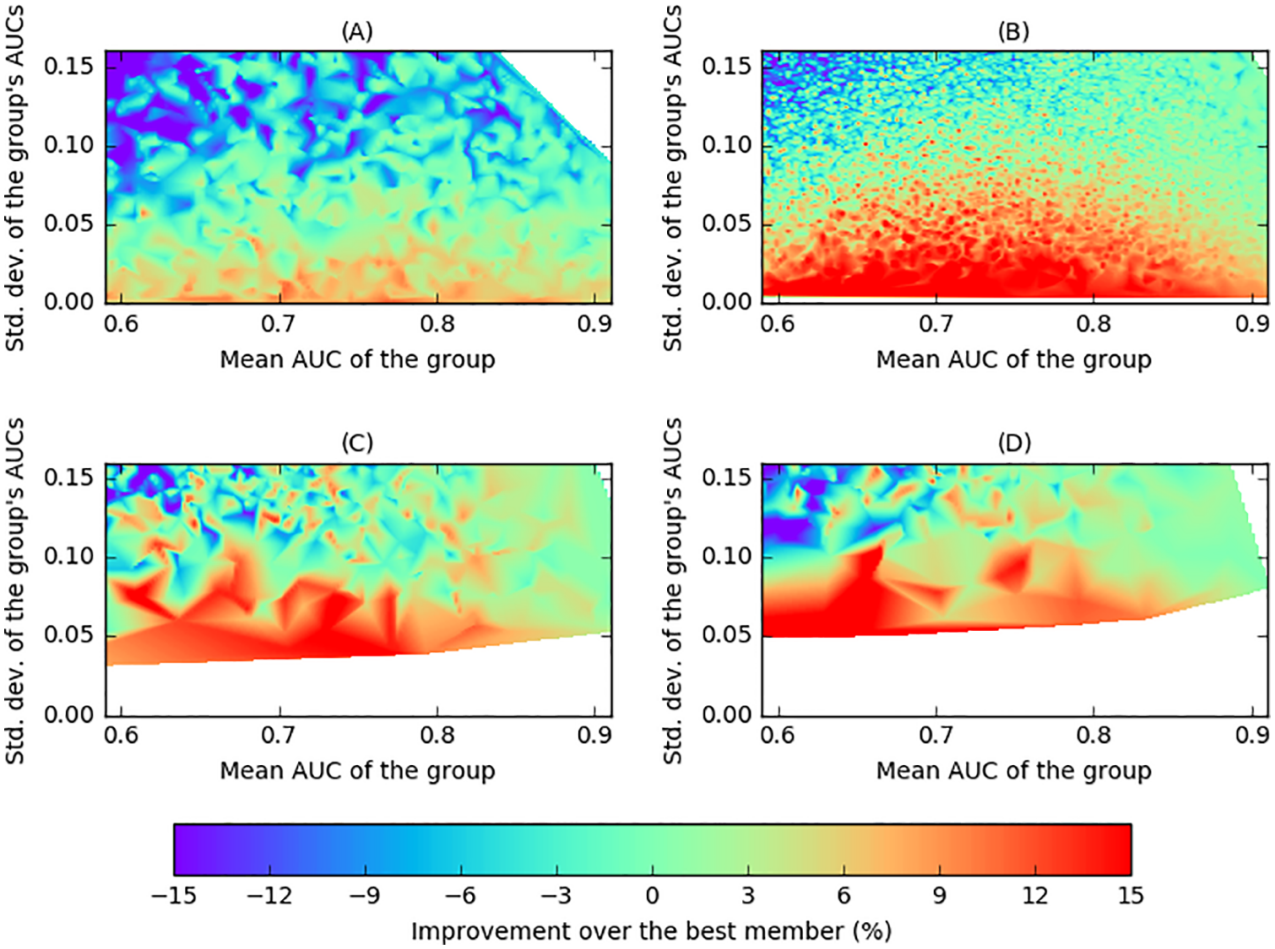
Surface interpolation of the expected changes in the AUC (in percentage) over the AUC of the best member of the group, for different group sizes, according to the theoretical model, when the distributions of scores for both classes are given by normally distributed random variables, $S_{i,c}∼N\left(\mu_{c},\sigma_{i}^{2}\right)$, with |*μ*_*C*_1_ = *L*_ − *μ*_*C*_2_ = *R*_| = 1 and standard deviations *σ*_*i*_ ∈ [0.3, 4]. (A) Size 2. (B) Size 4. (C) Size 6. (D) Size 8.

Fig 7A shows the expected changes in performance of pairs over the better participants predicted by this model under the assumptions above. The parameters for the Gaussian variables used in the simulations (i.e., |*μ*_*C*_1__ − *μ*_*C*_2__| = 1 and standard deviations *σ*_*i*_ ∈ [0.3, 4]) were estimated from the data collected from the experiment. The general trend using the proposed model is that there are gains (with respect to the AUC of the better participant) when the participants are similar (i.e., at the bottom of the figure), with the higher the similarity, the higher the gain. Even though there are differences between the theoretical predictions in this plot with the actual results in Fig 6A, the general similarity between the figures is striking, suggesting that a significant proportion of the effect is captured by the model.

Under the assumptions listed above, the model can easily be generalised to groups of size *r* > 2. In this case, the distributions of scores for a group, for each class, i.e., $S_{R,c}∼N\left(\mu_{R,c},\sigma_{R,c}^{2}\right)$, are determined by parameters

$$\begin{array}{c} \mu_{R,c}=\mu_{c}\;\;\;\;\text{and}\;\;\;\;\sigma_{R,c}^{2}=\sigma_{R}^{2}=\frac{\sum_{i\in R}\sigma_{i}^{2}}{r^{2}}, \end{array}$$

where *R* is the set of *r* participants included in the group. As before, in this case, the AUC resulting from the groups’ scores for each class will be higher than that of the best participant if $\sigma_{R}^{2}<\sigma_{min}^{2}$, with $\sigma_{min}^{2}=\min_{i\in R}\left(\sigma_{i}^{2}\right)$.

Fig 7 shows the expected changes in performance for groups of different sizes over the best participant of each group predicted by this model under the assumptions above. The figure illustrates the same trend as before (i.e., bigger gains are obtained at the bottom of the plots, corresponding to groups formed by participant with similar performance), and also matches to a significant degree the experimental results from previous sections, which are illustrated in Fig 6.

### Prediction of the analogue position of targets

We start this section by looking at the performance of single-user BCIs at predicting target position. For the sake of clarity to the reader, we will refer to this method as the single-user neural network-based BCI (sNN-BCI). Table 7 shows, for each presentation rate, the average Pearson’s correlation coefficient between the real and the predicted x-coordinate of the targets, across all participants, as well as the mean slope of a regression line fitted across the predictions.

**Table 7:** Mean and standard deviation of the correlation coefficient and the slope of the regression line fitted to the outputs of the sNN-BCI across all participants for each presentation rate.

Presentation rate	Correlation (*μ* ± SD)	Regression slope (*μ* ± SD)
5 Hz	0.19 ± 0.12	0.10 ± 0.07
6 Hz	0.26 ± 0.13	0.15 ± 0.09
10 Hz	0.19 ± 0.12	0.11 ± 0.07

**Fig 8:**
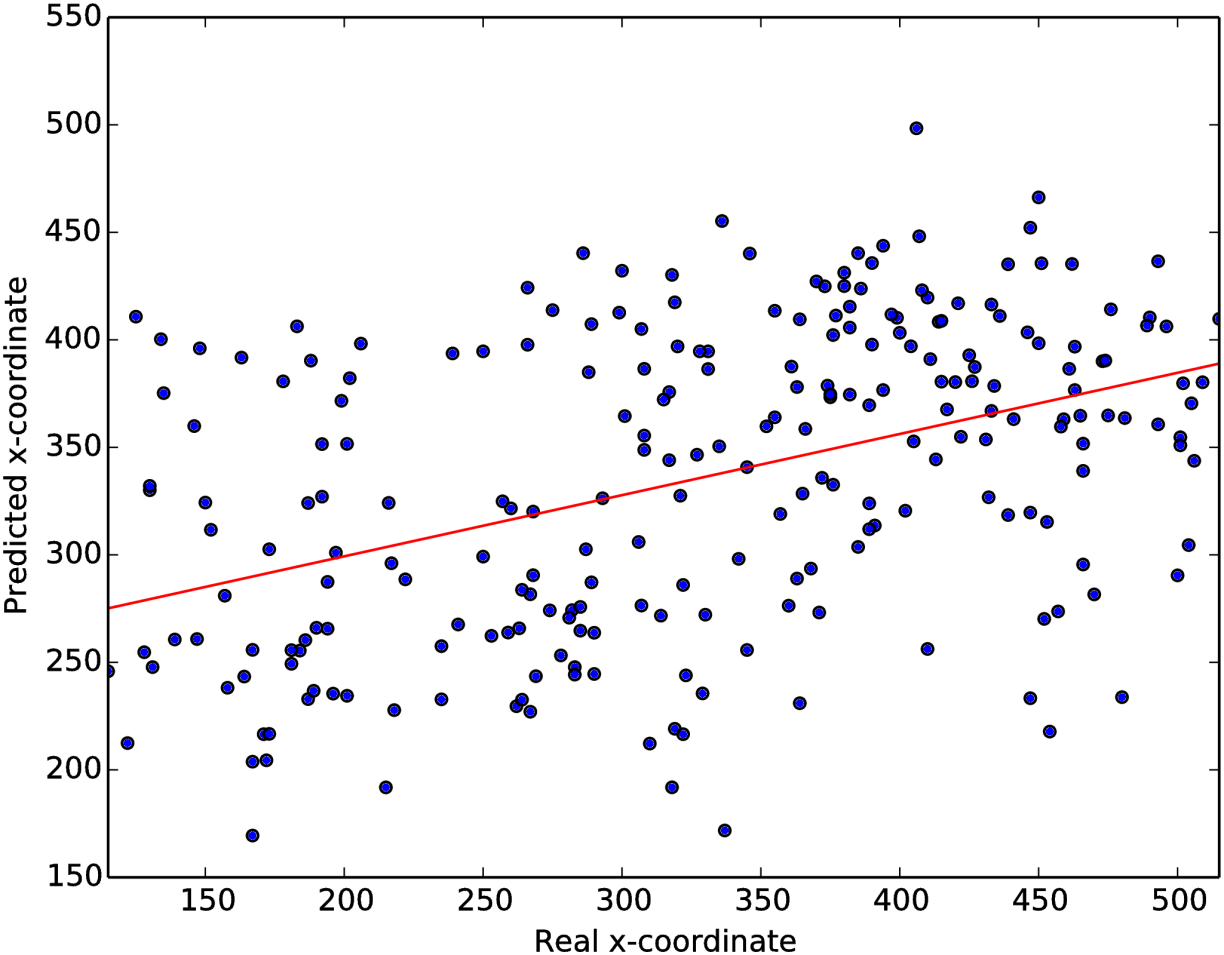
Predicted vs real x-coordinate of targets for the best performer at this task using the sNN-BCI (participant 3, 6 Hz).

Despite the low average correlation coefficients reported in the table, individual users can achieve much higher correlations. The highest, *ρ* = 0.43, was recorded by participant 3 at a presentation rate of 6 Hz, together with a regression slope *β* = 0.28 (the highest slope across all individuals and presentation rates). Fig 8 shows the predicted vs real coordinate of all target images in the test set for this participant and level. It is noticeable that, even for the best participant and level, the predictions of the system are not very accurate.

We now turn to the results obtained when using the collaborative approach at the output of the neural network (collaborative neural network-based BCI, cNN-BCI—see Collaborative target localisation section). Table 8 shows the mean and standard deviation of Pearson’s correlation coefficients for all presentation rates and group sizes. Similarly, the average regression slopes are reported in Table 9. In general, both the correlations and the regression slopes increase with group size. Moreover, as in the single-user case reported in Table 7, mean values are recorded at 6 Hz, and then decrease for the higher presentation rate of 10 Hz, showing the same behaviour that we observed for the peak amplitude of the N2pc ERP (see Fig 4).

**Table 8:** Mean and standard deviation of the correlation coefficient between actual and predicted x-coordinate of targets for different group sizes using the cNN-BCI approach.

Size	5 Hz	6 Hz	10 Hz
2	0.40 ± 0.09	0.44 ± 0.13	0.33 ± 0.14
3	0.46 ± 0.08	0.51 ± 0.10	0.40 ± 0.11
4	0.51 ± 0.07	0.56 ± 0.08	0.44 ± 0.09
5	0.54 ± 0.06	0.59 ± 0.07	0.47 ± 0.07
6	0.57 ± 0.05	0.62 ± 0.05	0.50 ± 0.06
7	0.59 ± 0.04	0.64 ± 0.04	0.51 ± 0.05
8	0.60 ± 0.04	0.66 ± 0.04	0.53 ± 0.04
9	0.62 ± 0.03	0.68 ± 0.03	0.54 ± 0.02

**Table 9:** Mean and standard deviation of the slope of the regression line fitted to the outputs of the cNN-BCI for different group sizes and presentation rates.

Size	5 Hz	6 Hz	10 Hz
2	0.37 ± 0.09	0.42 ± 0.14	0.31 ± 0.13
3	0.39 ± 0.07	0.45 ± 0.10	0.33 ± 0.10
4	0.40 ± 0.06	0.46 ± 0.08	0.34 ± 0.08
5	0.40 ± 0.05	0.47 ± 0.07	0.35 ± 0.07
6	0.41 ± 0.04	0.48 ± 0.05	0.35 ± 0.06
7	0.41 ± 0.03	0.48 ± 0.05	0.36 ± 0.05
8	0.41 ± 0.03	0.49 ± 0.04	0.36 ± 0.04
9	0.41 ± 0.03	0.49 ± 0.03	0.35 ± 0.02

As we will discuss below, the slope of the regression line and the correlation coefficient are closely related through the variance of the system outputs. Table 10 reports the ratios between the mean regression slopes and the mean correlation coefficients for each presentation rate and group size. The highest values are obtained, once again, for the presentation rate of 6 Hz.

**Table 10:** Ratio between the mean regression slope and mean correlation coefficients between the predicted and the actual x-coordinates of targets.

Size	5 Hz	6 Hz	10 Hz
2	0.93	0.95	0.94
3	0.85	0.88	0.83
4	0.78	0.82	0.77
5	0.74	0.80	0.74
6	0.72	0.77	0.7
7	0.69	0.75	0.71
8	0.68	0.74	0.68
9	0.66	0.72	0.65

**Fig 9:**
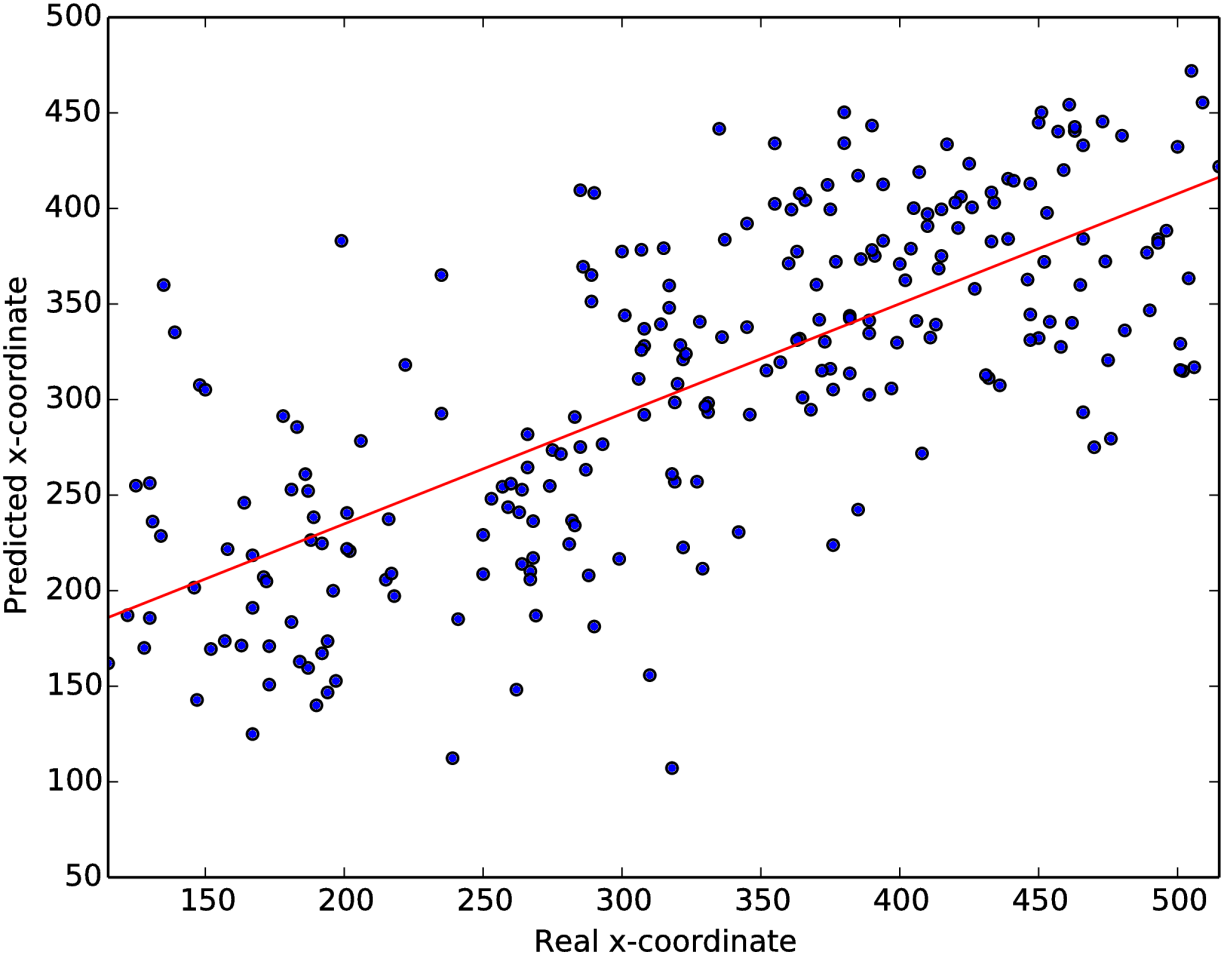
Predicted vs real x-coordinate of targets, for a group of size 7 at 6 Hz.

Fig 9 shows the predicted vs real coordinate of all target images in the test set of for one group of size 7 at a presentation rate of 6 Hz with a correlation coefficient of 0.72 (the highest obtained throughout all results) and a slope of 0.58. While this is again our top performer group, it is clear that through a cBCI predictions can be made much more accurately and are therefore of significantly higher practical utility.

## Discussion

In this paper we have used the N2pc component in single-user and collaborative BCIs to approximately locate targets in an RSVP paradigm, considerably extending previous research on this topic and opening a number of avenues for future work.

We started by analysing the timing and amplitude of the N2pc that is elicited in the conditions of our experiment, and studied three possible reasons for the changes in latency and amplitude that we found for the different presentation rates used in our protocol.

Although we were able to discard a possible refractory period of the N2pc as a reason for these rate-related variations, two other factors remain worthy of future exploration: (1) the fact that more and more targets were missed by participants as presentation rates increased; and (2) the experimental paradigm, that is, our decision not to randomise the order in which the levels of difficulty were presented based on participants not being able to cope with high presentation rates without prior habituation to the RSVP paradigm. In relation to the latter, after the standard practice sessions, participants were able to do reasonably well at the lowest presentation rate of 5 Hz, although in the early blocks many still lamented that the presentation rate was too fast. However, they progressively adjusted and later could cope with increases in the presentation rate.

Since the main purpose of the study was to demonstrate that collaborative BCIs can significantly improve the results obtained with single-user BCIs, not to establish whether they are best used at 5, 6 or 10 Hz, we felt that this was a reasonable compromise. In future research we will address this issue by adding a long pre-experiment practice session, e.g., by inviting participants twice: once for practice, and a second time, after they are rested again, for the real experiment. This will make it possible for participants to adapt to the speed of the RSVP protocol before the real experiment starts, thereby allowing a fully counterbalanced experimental design.

Of course, we cannot exclude the possibility that the two factors are related: when participants are tired (which in this case corresponds to the higher presentation rates, towards the final part of the experiment) they are more likely to perform badly in the visual search task. Moreover, we should not disregard the possibility that other unexplored factors are influencing the observed latency and amplitude of the N2pc.

In a first BCI, we used EEG data extracted from lateral targets to classify them into left visual field and right visual field targets, depending on whether they appeared on the left or the right side of an image. Our results (see Table 2) show large variations of performance across participants for this classification task, an effect that had been previously noted by Awni and collaborators [41], and our own previous research [29].

A reason for this large performance variations might be found in the choice of time window and electrodes. It should be noted that our choice of time window and electrode sites for extracting the epochs was based on previous literature and our own ERP analysis. We did not assess individual variability in N2pc latency or scalp distribution, which may be factors why these variations in performance occur. It is known that some ERPs, such as the P300, show high inter-subject variability [64]. However, the literature suggests that this is not the case for the N2pc ERP, whose latency is known to relate to different experimental paradigms or visual search tasks [33, 65, 66]. In the future, we intend to explore whether individually tailored time windows improve the performance of participants.

Another avenue for future work includes studying how and to which extent the airplane counts and classification performance are related, possibly with the addition of a P300-based BCI to detect targets. This study might also be helpful for determining whether lateral targets are more likely to be missed by participants as hypothesised in previous sections, and if this occurs more frequently at high presentation rates.

Regardless of the reported variations in individual performance, the AUC medians that we obtained from single-user BCIs are reasonably high [63], with performance for most participants being well above that of a random classifier (i.e., AUC = 0.5) and with the top quartile of our participants having AUCs ≥ 0.8. Overall, classification results indicate that target localisation by means of the N2pc is possible in the conditions of our experiments.

We used three different methods to combine information from users: one at the signal fusion level (the SC-cBCI approach) and two at the decision level (which we termed MC-cBCI and LDA-cBCI approaches). As expected from previous literature [5, 48, 49], the latter outperformed the signal fusion level. However, even the SC-cBCI method was capable of outperforming single-user BCIs.

By tabulating the results taking into account the similarity in performance of the participants that were used to form a group (see Table 4), we showed that performance increases dramatically when only participants with relatively similar performance are used. To establish a baseline, in this work, we first positioned ourselves in the simplest conditions by giving every member of a group equal weight (MC-cBCI approach). Of course, as a result of this, when the dissimilarity-index *δ* is high, good performers are dragged down by those participants who did not perform so well, so the gains are lower than for small values of *δ*. We then used an LDA classifier to intelligently assign different weights to the different members of a group, further increasing the average group performance.

The reduction in cBCI performance associated with high values of the dissimilarity index (which is represented in the top part of each plot of Fig 6) seems reasonable. For instance, in the case of groups of two individuals, when a high and a low performer are paired together (thus leading to a high dissimilarity index), the limited information provided by the low-performance individual with respect to that provided by the better performer is unlikely to provide an advantage for the latter (and indeed, it is likely to add noise to the decisions).

Under the assumption that single-user BCI performance is associated to the visual system sensitivity of an individual, we can link our results with those of Bahrami and collaborators [52], who showed that pairing participants based on the similarity of their visual sensitivities increased the performance with respect to randomly assigning observers to pairs. Despite the differences between their experimental protocol and ours (e.g., our participants were not able to communicate with each other), we have shown that the improvements in performance are higher when users are grouped using low values of our threshold *δ* (i.e., observers with similar visual sensitivities).

We developed a theoretical model that could explain the reasons for the higher improvements in the performance of our cBCI systems when groups are formed taking into account the similarity of the performance of the individuals in the group. Despite some simplifying assumptions made when developing this model, the results from our simulations show approximately the same behaviour as the experimental results, indicating that our model captures most of the reasons for the performance improvements of the cBCIs.

We did not study the effects of group selection in cBCIs for cortically-coupled vision (i.e., target detection) [6, 7], but, considering the generality of the assumptions that were made for the development of the theoretical model, we would expect that significant improvements could be obtained in such systems too. This remains as a task for future exploration.

In previous research [29] we showed that the outputs of the left vs right classifiers based on the N2pc are approximately correlated to the horizontal position of targets for the presentation rate of 5 Hz. In this paper we expanded on these results by increasing the presentation rates up to (and including) 10 Hz (cf. Fig 9), and studied the effect of group size on the correlation between x-coordinate of the target and its predicted coordinate, and on the slope of the linear regressor used for the prediction.

The correlation coefficient *ρ* and the regression slope *β* are known to be related through the following equation:

$$\begin{array}{c} \beta =\rho \times \frac{\sigma_{output}}{\sigma_{input}}, \end{array}$$

where *σ*_*output*_ and *σ*_*input*_ are, respectively, the standard deviations of the output (i.e., the values obtained from the linear regressor) and the input (in this case, the real x-coordinates of the targets) values. In the case of standardised variables, *β* = *ρ*. However, in all other cases, *ρ* and *β* give different information about the strength of the linear relationship between inputs and outputs: the correlation coefficient is independent of the scale of the variables, and gives information about how close they are to a perfect linear relationship; the regression slope is the change in the expected value of the outputs that corresponds to a change of one unit in the inputs.

In the proposed system, given that the inputs are always the same (and correspond to the x-coordinates of the targets in the RSVP experiment), changes in $\frac{\sigma_{output}}{\sigma_{input}}$ will effectively reflect changes in the variance of the outputs.

Considering this, the low slopes recorded in Table 7 for the sNN-BCI are an indication of the smaller variance of the predictions than the variance of the x-coordinates that are given as inputs. Indeed, the ratio $\frac{\beta }{\rho }$ is maintained around 0.5 across all levels.

In the collaborative case, interestingly, this ratio decreases with increasing group sizes, revealing that the standard deviation of the outputs decreases (with respect to the *constant* standard deviation of the inputs), as reported in Table 10, although it remains much higher than in the single-user case. The higher ratio shown by cNN-BCIs is a good thing in terms of the desired behaviour of the system, and, while it decreases for larger groups, it is still much better than for the single-user case.

Even though we have not studied the effects of applying the group selection method to the cNN-BCI approach, we noticed that some groups showed correlation coefficients and regression slopes much greater than the average, indicating that a group-member selection process could lead to much improved accuracy in this area too. More research will be devoted to participation selection processes in the future.

As a final remark, we would like to point out the fact that the results reported in this paper are derived from *offline* experiments only. We are aware of the need for testing the online performance of our system in future work, although we are cautiously optimistic considering that other groups have done online experiments involving the N2pc with good results [41]. Of course, online performance will depend on the choice of feedback given to users during online operation. If, for example, feedback is only given at the end of a burst, which is a reasonable choice for this type of application, we would expect performance to remain similar to the one obtained here.

Last, but not least, although it is not the norm, previous studies have shown that online systems might outperform results from offline experiments using the P300 component [39, 67]. Although the reasons behind this remain unknown, this might be due to higher subject engagement in the experiment, or a participant’s desire to self-improve. Although we cannot claim that the results of an online cBCI exploiting the N2pc will follow this behaviour, similar effects (e.g., motivation, etc. in online systems) would seem to be applicable to our setup.

## Conclusions

In this paper we used the N2pc ERP to establish, via both single-user and collaborative BCIs, the approximate location (along the horizontal axis) of targets in images shown at high presentation rates.

Firstly, we found that real-world target stimuli produce a distinctive N2pc at all presentation rates considered, and that the amplitude and latency of the N2pc evoked in our experiment change as the presentation rate is varied. We also analysed the potential sources for such variations, confirming that these are *not* due to a “refractory period” behaviour of the visual system.

We showed that it is possible to reliably detect the N2pc and use it to classify targets from real-world stimuli into LVF and RVF even in single-trial, single-user BCIs for presentation rates of up to 10 Hz. Moreover, by using simple methods for combining classifiers’ outputs, we also found that collaborative BCIs significantly outperform single-user BCIs in the left vs right classification task.

Even though this happens even when no group-member selection is applied, performance increases dramatically when only participants with relatively similar performance are used to form a group. We developed and tested a theoretical model that could explain the reasons for this behaviour. By comparing the results from our simulations with the experimental results, we established that our model captures most of the reasons for the performance improvements of the cBCIs.

Cortically-coupled vision, so far, has focused on the task of target detection in image triage by means of the P300. Here, we looked at identifying the position of targets. We believe that future research in this area of application should explore ways of combining both systems, now that it has been established that both the P300 and the N2pc can be detected independently. One possible way of achieving this is by cascading the two classifiers: after the P300-based target detection mechanism decides that a given image contains a target, the N2pc-based left vs right classifier could help locate the side of the image where the target is (or even provide a rough idea of its position). In this way, current cortically-coupled vision or triage systems could be improved to reduce the (current) effort needed to manually locate targets after their detection.
